# Retrospect and Risk Analysis of Foot-and-Mouth Disease in China Based on Integrated Surveillance and Spatial Analysis Tools

**DOI:** 10.3389/fvets.2019.00511

**Published:** 2020-01-21

**Authors:** Jiahui Chen, Jianying Wang, Minjia Wang, Ruirui Liang, Yi Lu, Qiang Zhang, Qin Chen, Bing Niu

**Affiliations:** ^1^Shanghai Key Laboratory of Bio-Energy Crops, School of Life Sciences, Shanghai University, Shanghai, China; ^2^Tech Ctr Anim Plant & Food Inspect & Quarantine, Shanghai Customs, Shanghai, China

**Keywords:** foot-and-mouth disease (FMD), spatial statistics, standard deviational ellipse, space-time scan statistics, hotspot detection, geographic information system (GIS)

## Abstract

Foot-and-mouth disease (FMD) is a highly contagious disease of livestock and seriously affects the development of animal husbandry. It is necessary to defend the spread of FMD. To explore the distribution characteristics and transmission of FMD between 2010 and 2017 in China, Global Moran's I test and Getis-Ord Gi index were used to analyze the spatial cluster. A space-time permutation scan statistic was applied to analyze the spatio-temporal pattern. GIS-based method was employed to create a map representing the distribution pattern, directional trend, and hotspots for each outbreak. The number of cases was defined as the number of animals with FMD for the above analysis. We also constructed a phylogenetic tree to compare the homology and variation of FMD virus (FMDV) to provide a clue for the potential development of an effective vaccine. The results indicated that the FMD outbreaks in China had obvious time patterns and clusters in space and space-time, with the outbreaks concentrated in the first half of each year. The outbreaks of FMD decreased each year from 2010 with an obvious downward trend of hotspots. Spatial analysis revealed that the distribution of FMD outbreaks in 2010, 2015, and 2017 exhibited a clustered pattern. Space-time scanning revealed that the spatio-temporal clusters were centered in Guangdong, Tibet and the junction of Wuhan, Jiangxi, Anhui. Comparison of the spatial analysis and space-time analysis of FMD outbreaks revealed that Guangdong was the same cluster of the two in 2010. In addition, the directional trend analysis indicated that the FMD transmission was oriented northwest-southeast. The findings demonstrated that FMDV in China can be divided into three pedigrees and the homology of these strains is very high while comparing the first FMDV strain with the others. The data provide a basis for the effective monitoring and prevention of FMD, and for the development of an FMD vaccine in China.

## Introduction

Foot-and-mouth disease (FMD) is one of the most contagious diseases of cloven-hoofed animals (1), including cattle, swine, sheep, and goats, as well as many species of wild animals (2). FMD virus (FMDV) is a non-enveloped RNA virus belonging to the genus Aphthovirus of family Picornaviridae (3), which is divided into seven serotypes (A, O, C, Asia 1, SAT1, SAT2, and SAT3) based on their serological relationship (4). Infected animals are characterized by fever, lameness, painful sores in the mouth, and blisters on the tongue, feet, nares, muzzle, and teats. The pain and discomfort from the lesions leads to depression, anorexia, lameness, and reluctance to move, which causes severe losses in animal production (5, 6). FMD impacts on animal production include reduced milk yields, loss of weight in growing animals, and reduced ability to work in terms of cultivation and transporting goods (7). FMD has a high morbidity, which has prompted governments to impose restrictions on the trade of animals locally and internationally.

**Figure 1:**
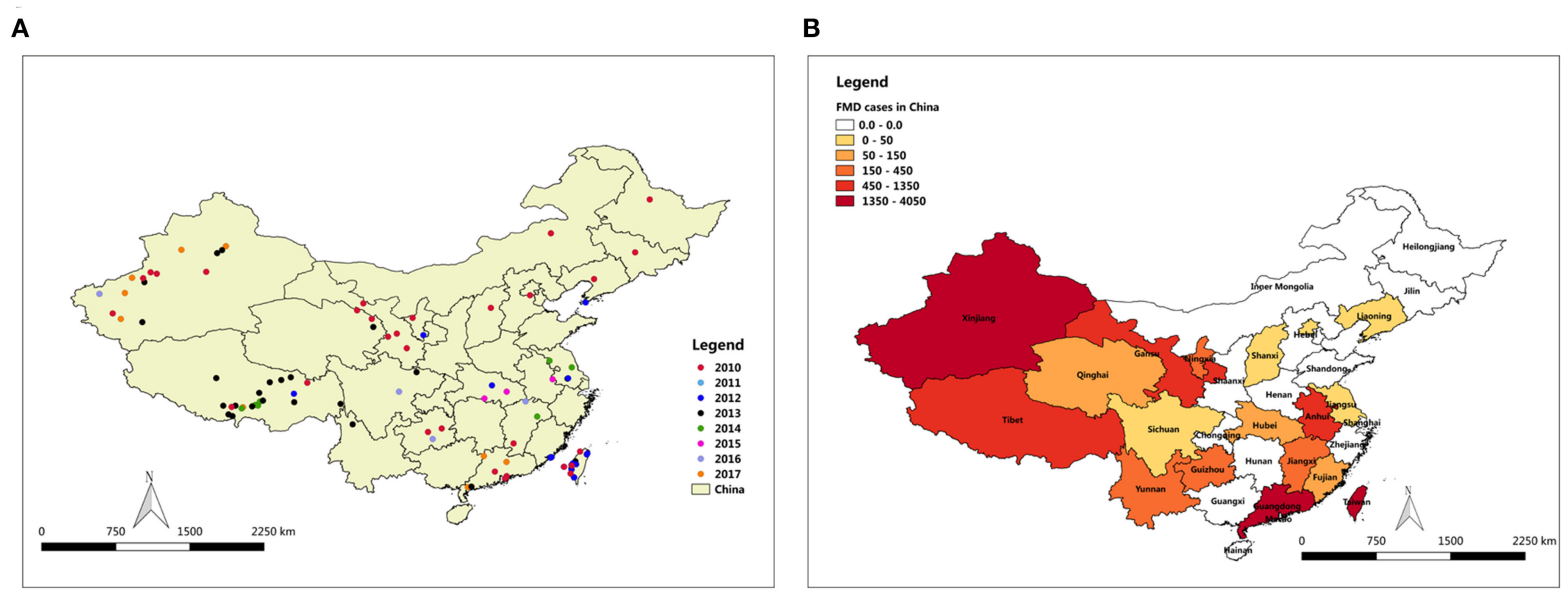
Map of FMD outbreaks in China from 2010 to 2017. **(A)** FMD affected locality in China, **(B)** FMD cases degree in all provinces of China.

FMD has been reported in many countries. Outbreaks have been concentrated in Asia, Africa and parts of Europe adjacent to Asia (8). Prior to 1997, countries in eastern Asia, such as Taipei China, Japan, and Republic of Korea, had been free from FMD for decades (9). In China, the outbreak of FMD cases has been declining for the past 8 years. The distribution map of FMD reveals that the outbreaks of FMD were focused in northwest and southeast of China, including Taiwan (29 outbreaks with 2,064 cases), Tibet (26 outbreaks with 1,056 cases), and Xinjiang (19 outbreaks with 1,917 cases) (Figure 1). The large number of cases in these outbreaks of FMD was related to the high population density of susceptible species in these areas. For example, in 2017, the total stock of cattle, sheep, and swine in Xinjiang was 50.9 million, and the total stock of Tibetan cattle, sheep, and swine was 17.4 million.

The characteristics calculated by spatial cluster analysis provide an important basis for the prevention and control for FMD (10). According to OIE reports, geographic areas, and populations at the highest risk are very significant in the battle against infectious diseases (11). In previous studies, spatial statistics analysis as a method is more inclined to combine with other methods to improve the ability of spatial distribution analysis approach and the description of disease dynamics transmission. Getis-Ord Gi statistic and standard deviational ellipse as classical spatial analysis methods are often used for disease hotspots analysis and directional trend identification of disease outbreaks. For example, Ahmet et al. applied local Moran's I and local Getis-Ord Gi ^*^(d) statistics to identify the localities with clusters and hot spots of Hepatitis A abundance in 2001, 2006, and 2011 (12). Ratchaphon et al. utilized standard deviational ellipse to identify the trend of the global diffusion pattern of the affected villages (13).

Space-time permutation model as another common spatial tool was also used in the description of spatial and temporal dynamics changes of FMD outbreaks (14, 15). Abdrakhmanov et al. built a spatio-temporal analysis model to describe and analyze the changes in spatial and temporal dynamics of FMD under different control strategies in the Republic of Kazakhstan from 1955 to 2013 (16). Souley et al. utilized the spatio-temporal patterns of transmission of FMD outbreaks in Niger to improve knowledge on the disease dynamics and factors related to FMD occurrence and conduct an efficient control (17). Jemberu et al. used a retrospective analysis to determine the incidence, distribution, risk factors, and causal serotypes of FMD outbreaks in Ethiopia (18).

In the current study, Global Moran's I test and heat maps were utilized to investigate the distribution characteristics based on the collection of FMD data in China from 2010 to 2017. Space-time permutation scan statistic was applied to analysis spatio-temporal pattern. Furthermore, the monthly trends of FMD outbreaks were obtained by analyzing the prevalence of FMD. A hotspot distribution map representing each outbreak was drawn based on the calculated significance of FMD hotspots. Directional distribution analysis were performed to depict the directional trend of FMD outbreaks and to find out the direction of disease transmission.

These findings provide valuable information for monitor, prevention and control, and of FMD. After analyzing the distribution characteristics and transmission routes of FMD in China, a phylogenetic tree was constructed to analyze the homology and variation of FMDV genomes. This data will help in the development of an effective FMD vaccine.

## Methods

### Data Acquisition

FMD data including affected animal species and the number of cases, as well as the geographical location of the epidemic regions, were collected from Global Animal Disease Information System (EMPRES) in Food and Agriculture Organization of the United Nations (FAO) (http://empres-i.fao.org/eipws3g/#h=0, 2019). A case is the number of animals suffering from FMD. The number of cases at the same geographical location is considered as an outbreak point, with the same outbreak ID in EMPRES. EMPRES-i organizes all available epidemiological data on specific animal disease events as comprehensive records and assigns individual event identifiers (id).

### Two-Dimensional Standard Deviational Ellipse (SDE)

SDE is a spatial statistical tool that is generally used to depict the directional trend of the geographical features of the spatial distribution (19). Working operation of SDE has been described in detail in ArcGIS Help 10.1 and Wang et al. (20, 21).

### Hotspots Analysis

Hotspots analysis is valuable for identifying spatial clusters. The analysis uses the Getis-Ord Gi statistics to detect the statistically significant high values (hot spots) and low values (cold spots). Hotspots are spatial clusters with high correlation within a specified distance of the entire research area identified by the Getis-Ord Gi statistics. Hotspot detection is sensitive when clustering local spatial events if there is no significant global spatial clustering (22). Getis-Ord Gi^*^ statistics was calculated to test statistical significance of spatial clustering patterns like hot spots, random spots, and cold spots over the entire study area. Getis-Ord Gi^*^ statistics formula refers to the method of Ord et al. (23). The window width was enforced to determine the boundaries of the hot and cold spots in the spatial clusters.

### Global Spatial Autocorrelation

Global Moran's I statistic as a spatial-correlation statistic was used to assess the presence of significant spatial autocorrelation and find the characteristics of the global pattern (clustered, dispersed, random) of FMD (24, 25). Moran's I Index varies between +1 to −1, a positive value (Moran's I Index > 0) indicates the presence of positive correlation in spatial distribution, while a negative value (Moran's I Index < 0) indicates presence of negative correlation in the spatial distribution. The larger the Moran's I Index value, the more obvious of positive correlation. When Moran's I Index = 0 indicates a random spatial distribution (26). The Moran's I formula refers to Guo et al. (27).

### Spatio-Temporal Cluster Analysis

To identify the spatio-temporal clusters of FMD outbreaks, a retrospective permutation space-time scan model was developed in the SaTScan environment. The method was applied by building a space-time cylinder to scan the study area by placing a number of circles (spatial windows). The radius at the bottom of cylinder represents geographic position and size of cluster area. The height of cylinder denotes time of outbreaks (28). As the constant changes of radius and time, spatial window is in dynamic changing, and counting the number of positive and/or negative events occurring (29).

### Phylogenetic Analysis of FMDV Genomes

Thirty-one registered FMDV genome sequences were obtained from GenBank. The phylogenetic tree of FMDV was constructed by the Neighbor-Joining method and the Kimura 2-parameter model of MEGA6 software. The Boot-strap validation complex is 1,000, and the branch of phylogenetic tree whose Boot-strap value is more than 70% is considered high reliability (30, 31).

## Results

### Prevalence of FMD

**Figure 2:**
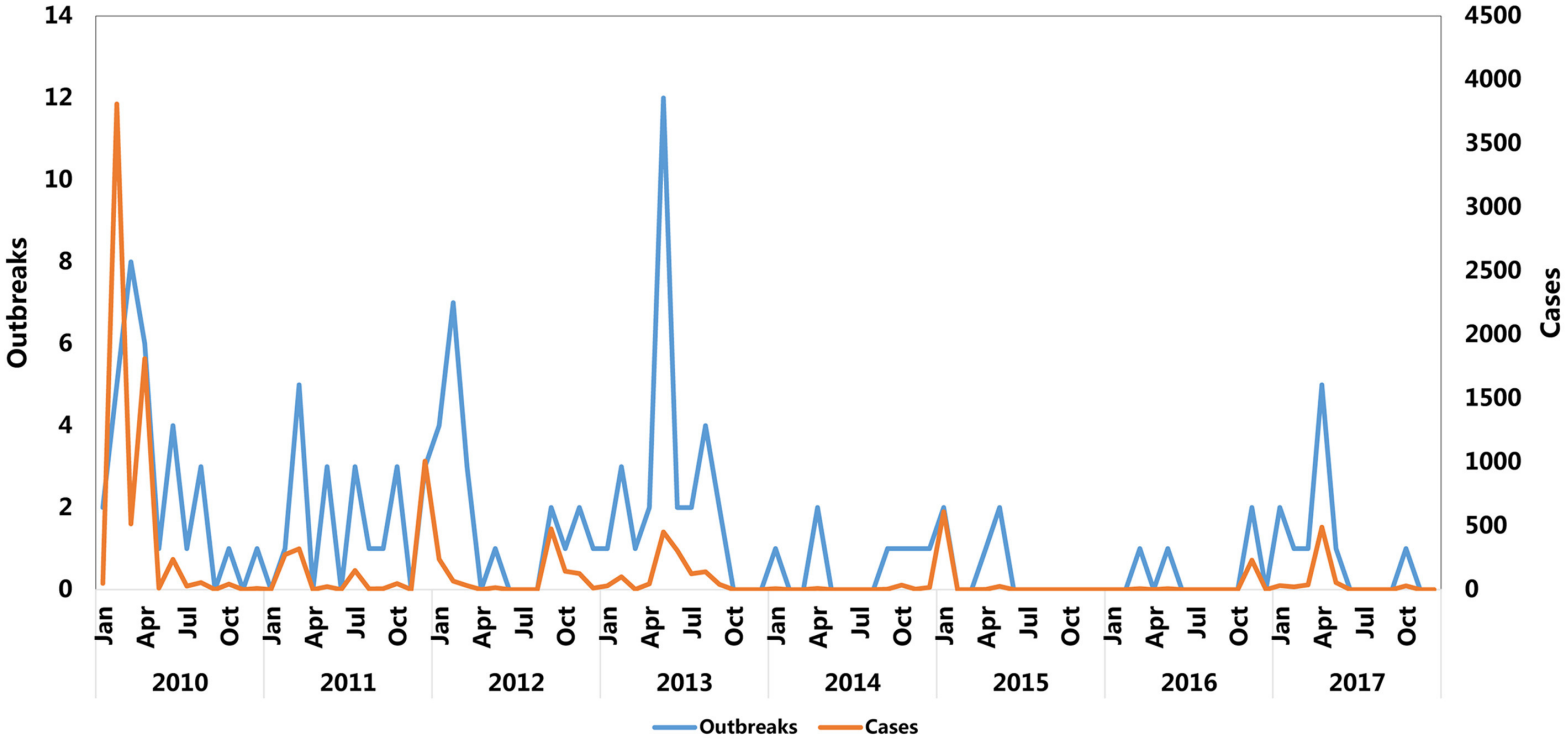
Trend of monthly FMD cases from 2010 to 2017.

The monthly reported cases and FMD outbreaks of China from 2010 to 2017 are summarized in Figure 2 and Table 1. The FMD outbreaks gradually decreased. The frequency of outbreaks and cases of FMD before 2010 to 2013 are higher than 2014 to 2017. Except for September, August, October, and November, the number of cases in other months reached more than 500. The highest peaks of infections per year occurred in different months and FMD outbreaks were concentrated in the first half of the year. February, April and December were the peak months of FMD outbreaks, in which there were 17, 16, and 6 outbreaks, respectively. The respective number of cases was 4,273 (*P* < 0.001), 2,355 (*P* < 0.001), and 1,047 (*P* < 0.001).

**Table 1:** Monthly FMD outbreaks and cases from 2010 to 2017.

**Months**	**2010**	**2011**	**2012**	**2013**	**2014**	**2015**	**2016**	**2017**	**Sum**	**Mean**
	**Outbreaks**									
Jan	2	0	4	1	1	2	0	2	12	1.50
Feb	5	1	7	3	0	0	0	1	17	2.13
Mar	8	5	3	1	0	0	1	1	19	2.38
Apr	6	0	0	2	2	1	0	5	16	2.00
May	1	3	1	12	0	2	1	0	20	2.50
Jun	4	0	0	2	0	0	0	0	6	0.75
Jul	1	3	0	2	0	0	0	0	6	0.75
Aug	3	1	0	4	0	0	0	0	8	1.00
Sep	0	1	2	2	1	0	0	1	8	1.00
Oct	1	3	1	0	1	0	0	1	7	0.88
Nov	0	0	2	0	1	0	2	0	5	0.63
Dec	1	3	1	0	1	0	0	0	6	0.75
**Months**	**Cases**								**Sum**	**Mean**
Jan	49	0	239	30	7	610	0	32	967	120.88
Feb	3810	275	66	101	0	0	0	21	4273	534.13
Mar	516	319	32	2	0	0	8	37	914	114.25
Apr	1812	0	0	44	9	1	0	489	2355	294.38
May	12	24	15	452	0	26	7	55	591	73.88
Jun	236	0	0	305	0	0	0	0	541	67.63
Jul	28	150	0	124	0	0	0	0	302	37.75
Aug	55	6	0	140	0	0	0	0	201	25.13
Sep	0	7	476	40	3	0	0	0	526	65.75
Oct	42	46	144	0	34	0	0	30	296	37.00
Nov	0	0	126	0	4	0	230	0	360	45.00
Dec	10	1008	12	0	17	0	0	0	1047	130.88

**Figure 3:**
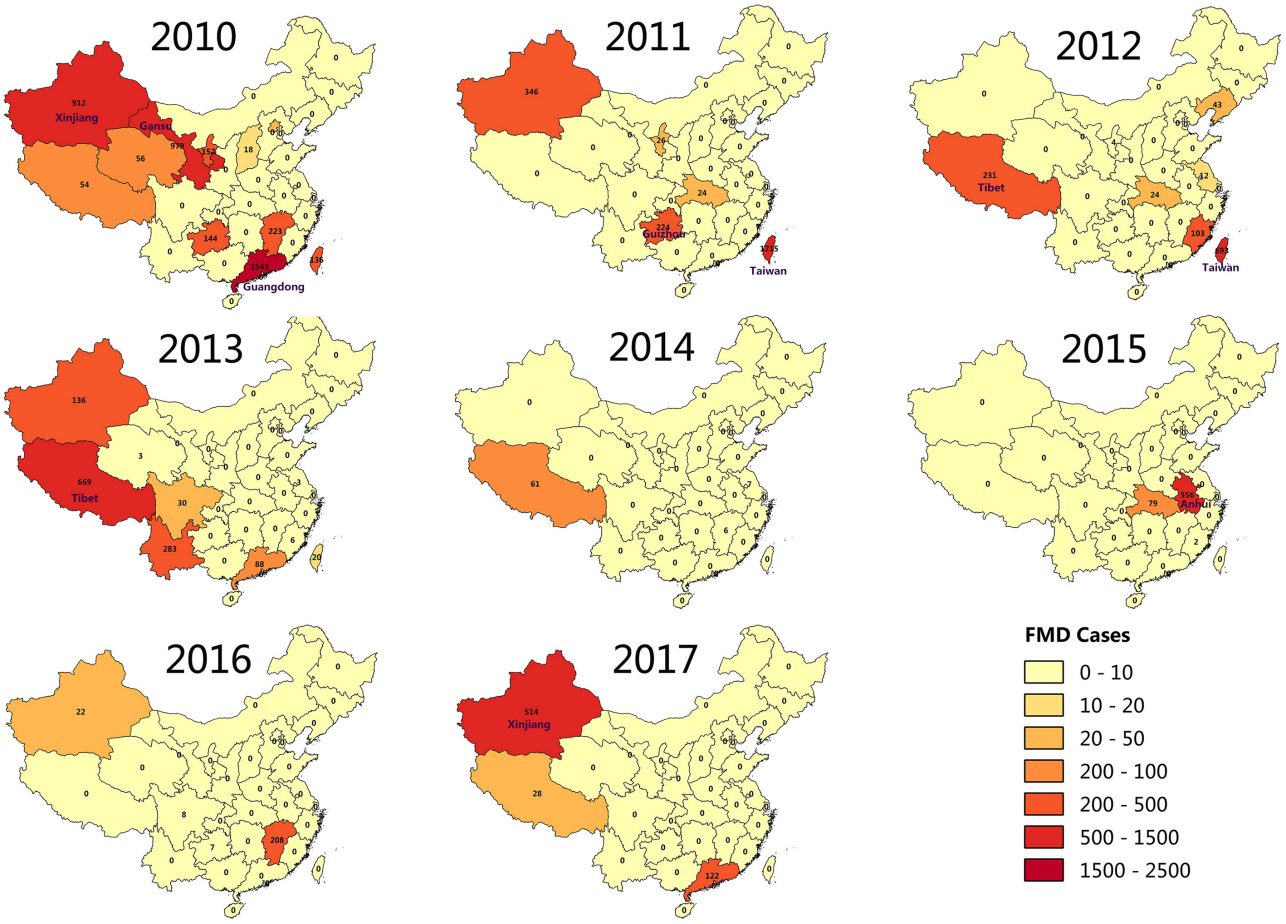
FMD cases in china, annually from 2010 to 2017.

FMD outbreaks occurred in several provinces of China during 2010 to 2017. To further explore the annual FMD cases density of various provinces in China, a FMD cases density map was constructed by analyzing the relationship between annual FMD cases and affected localities (Figure 3). FMD was concentrated in the northwest region and Taiwan province of China from 2010 to 2017. Surprisingly, Xinjiang experienced FMD almost every year, except 2012, 2014, and 2015. Even in 2010, there were 5 outbreaks with 912 cases of FMD in Xinjiang. In 2013, 16 outbreaks with 669 cases of FMD were reported in Tibet. In addition, FMD appeared in Taiwan for 4 years from 2010 to 2013. In 2011, there were 12 outbreaks of FMD in Taiwan with 1215 cases reported. In 2010, FMD outbreaks in Guangdong and Hong Kong were more serious, with 1,543 and 2,330 cases, respectively.

### Hotspot Detection and Analysis

**Figure 4:**
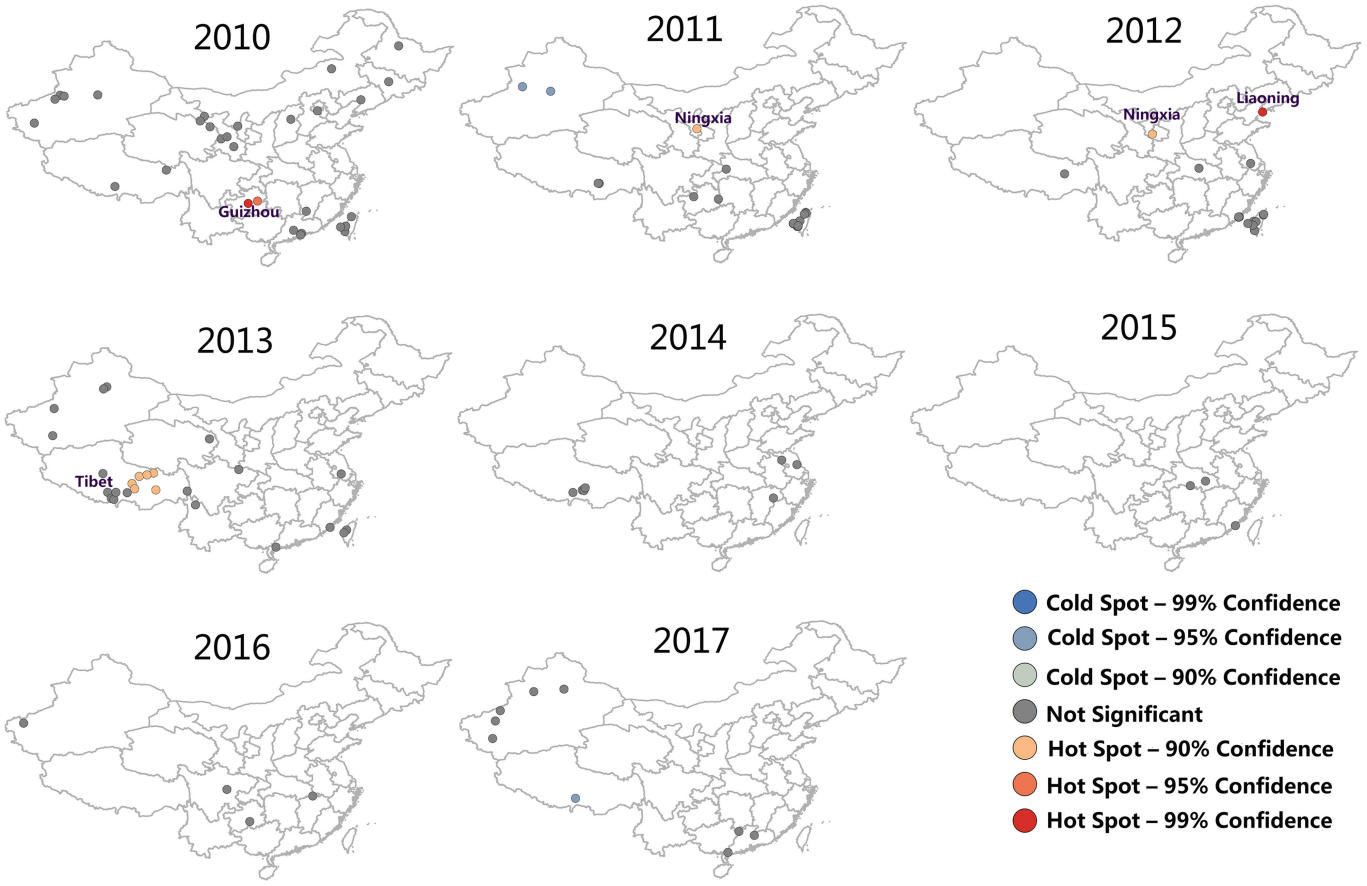
FMD hotspot development from 2010 to 2017. The red dot were represented hotspot (high FMD outbreaks clustered), the blue dot were represented coldspot (low FMD outbreaks clustered), and the gray dot indicate there was no significance of this dot.

After conducting FMD hotspots analysis on Getis-Ord Gi, a map representing the significance of FMD outbreaks hotspots was prepared (Figure 4). The red dot and blue dot were represented hotspot (high FMD outbreaks clustered) and coldspot (low FMD outbreaks clustered), respectively, and gray dot indicate there was no significance of this dot. It can be found that FMD hotspots were dominantly clustered in Guizhou (2010), Ningxia (2011, 2012), Liaoning (2011), and Tibet (2013). In 2010 and 2012, there were significant hotspots with 99% confidence (*P* = 0.01) located in Guizhou and Liaoning, respectively. In 2017, there was a cold spot with 99% confidence of an FMD outbreak in Tibet.

**Figure 5:**
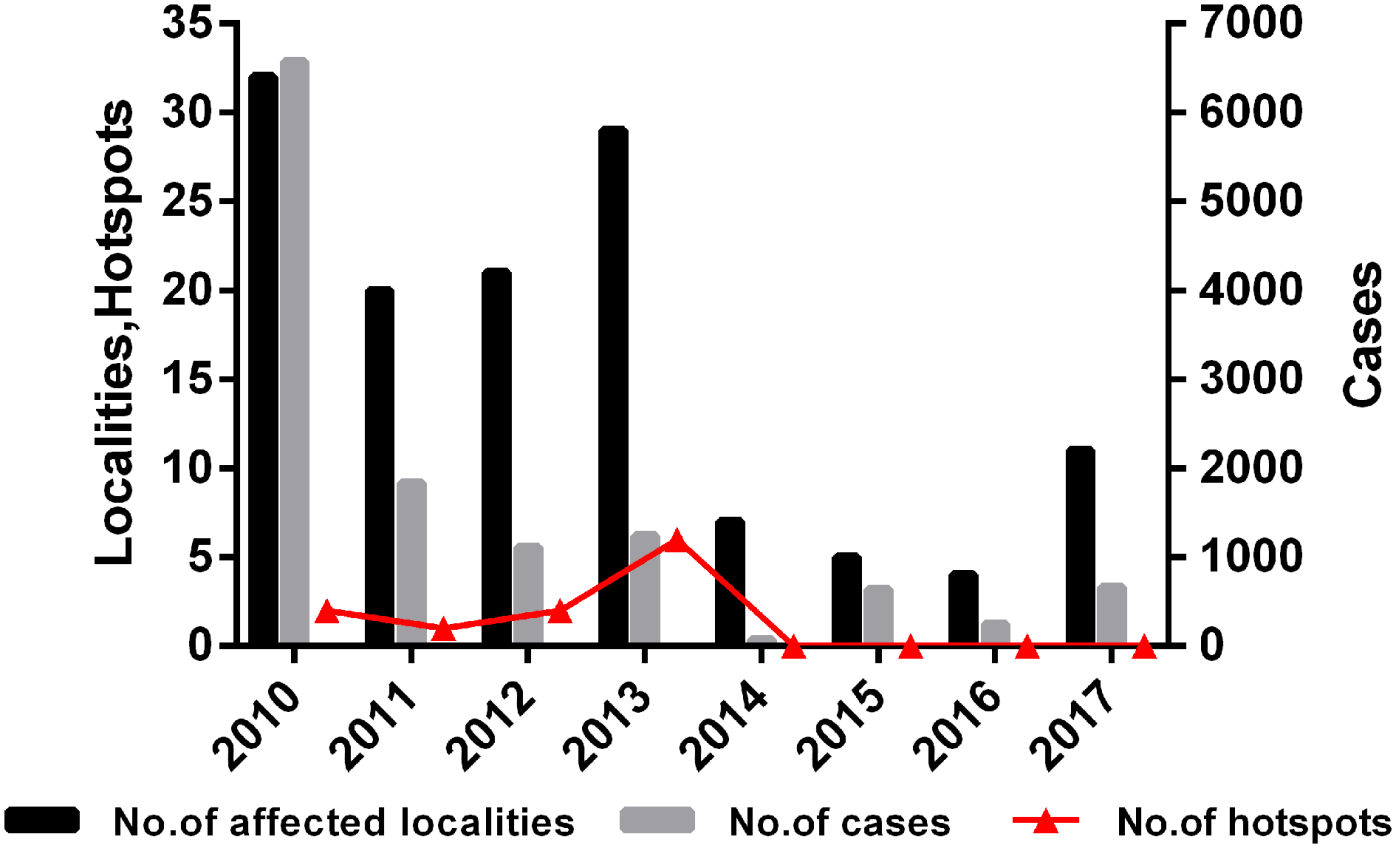
Number of affected localities, number of cases, and number of hotspots in China during 2010–2017.

In addition, the trend of affected localities, FMD cases and hotspots were shown in Figure 5. The results showed that the trends of the three types of data are very similar, which means there may be a correlation between hotspots trend of FMD and the actual outbreak situation. Hotspots increased from 2010 to 2013, and reached the peak in 2013 (6 hotspots in Tibet). There were fewer hotspots in 2013 with an ultimate decline to zero. The results indicated that the outbreaks of FMD decreased each year and that FMD hotspot analysis was instructive for the determination of the spatial distribution of FMD outbreaks.

### Spatial Distribution Pattern

**Figure 6:**
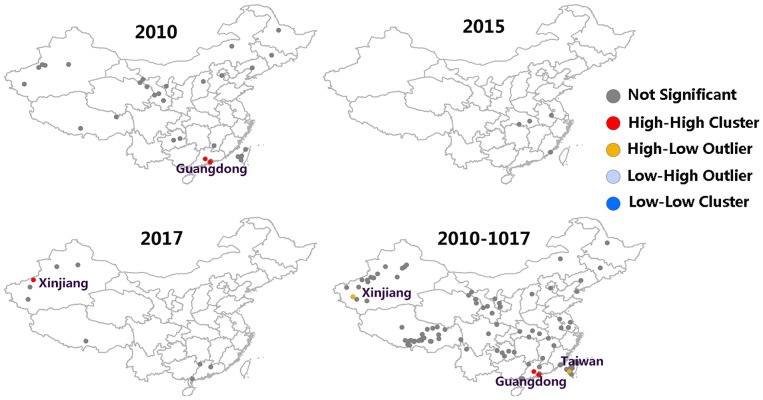
LISA cluster map of FMD in China.

**Figure 7:**
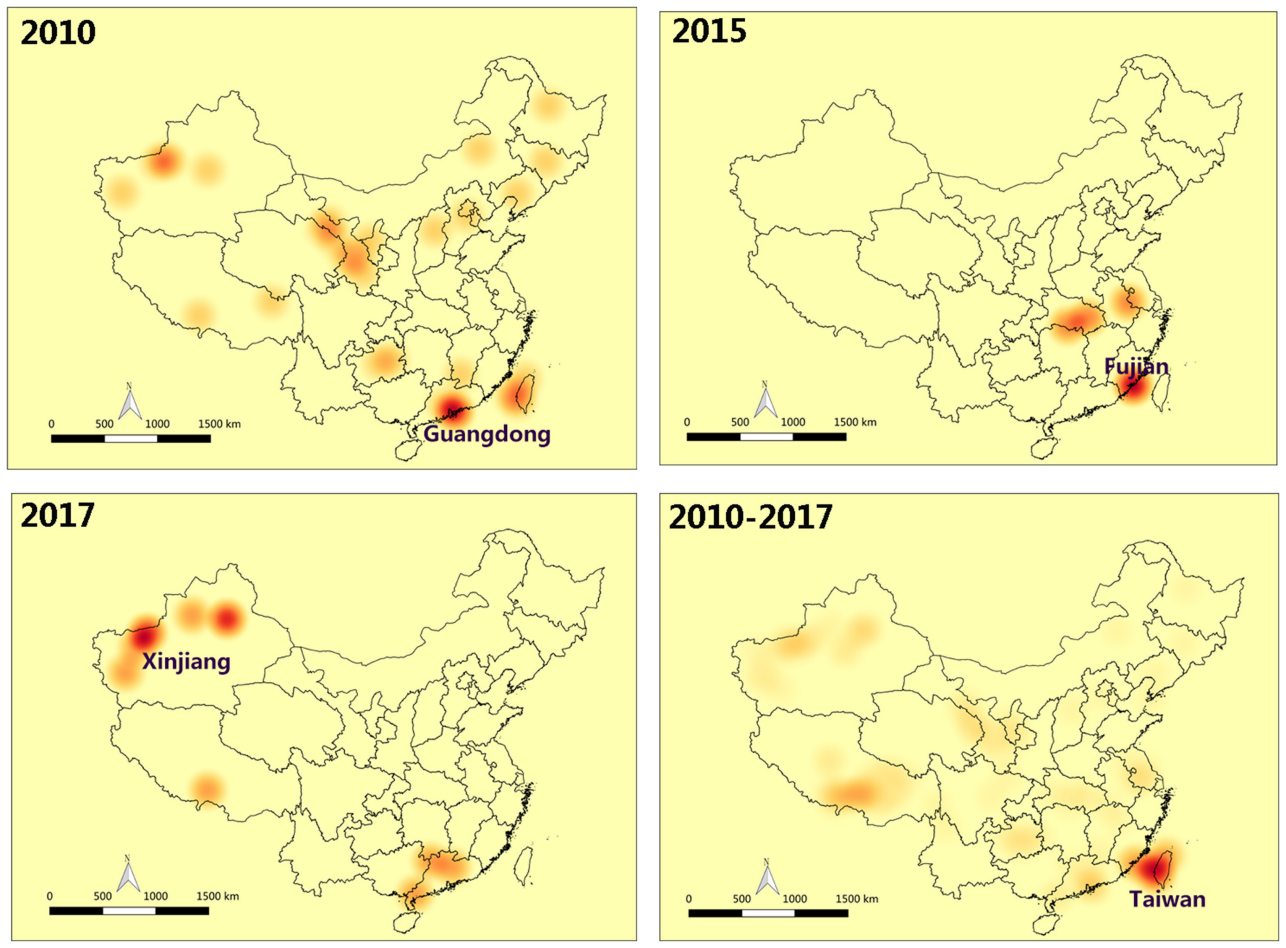
Heat map of FMD clusters in China. The dot of heat map indicates relative intensities of FMD clusters. Outermost (yellow): The low intensity of FMD outbreaks, middle (orange): The medium intensity of FMD outbreaks, innermost layer (dark red): The high intensity of FMD outbreaks.

Moran's I index is commonly used in spatial cluster analyses, which can represent a significant spatial auto-correlation of a study area. Here, Moran's I index significance test of FMD from 2010 to 2017 was calculated by the spatial autocorrelation statistical model. Moran's I index in 2010, 2015, and 2017 were positive (Moran's I index > 0) that exhibited a clustered tendency in spatial pattern (Table 2). In 2010, the Moran's I = 0.51, *Z*-score was 4.70 (≥1.96), which showed strong positive correlation in the spatial pattern. The probability of randomly generating a clustering pattern were less than 0.01 (*P* < 0.01) in 2010, probability in 2015 and 2017 were less than 0.1(*P* < 0.1) and 0.05 (*P* < 0.5) respectively. As there is no significant difference between the distribution patterns of FMD outbreaks and random patterns (in 2011–2014 and 2016), it could be regarded as a relatively unstable random distribution. Cumulative data from 2010 to 2017 showed that the spatial pattern of FMD outbreaks was significantly clustered. Hotspot analysis was used to further analyze the spatial clustering of FMD in China. A local spatial autocorrelation (LISA) cluster map showed high-high cluster points were located in Guangdong in 2010 and Xinjiang in 2017. However, there was no FMD cluster in 2015 (Figure 6). To explore whether the high-high spatial clusters were consistent with the high intensities of FMD cases in real outbreaks, FMD heat map of cluster year was created (Figure 7). The dot of heat map indicates relative intensities of FMD clusters. Outermost (yellow), middle (orange), and innermost layer (dark red) represented low, medium and high intensity FMD outbreaks, respectively. FMD outbreak clusters in the heat map and the LISA map were nearly superimposable in their positions. Especially, the high intensities of FMD cases dominantly clustered in Xinjiang, Qinghai, Tibet, Taiwan, and Guangdong.

**Table 2:** Global spatial autocorrelation analysis of FMD cases in China from 2010 to 2017.

**Years**	**Moran's I**	***Z*-score**	***P*-value**	**Pattern**
2010	0.51	4.7	<0.001	Clustered
2011	−0.13	−1.06	0.29	Random
2012	−0.02	0.59	0.56	Random
2013	−0.06	−0.38	0.71	Random
2014	−0.16	0.01	0.99	Random
2015	0.16	1.67	0.09	Clustered
2016	−0.31	0.08	0.94	Random
2017	0.31	2.02	0.04	Clustered
2010–2017	0.091	4.07	<0.001	Clustered

### Spatio-Temporal Cluster Analysis

**Figure 8:**
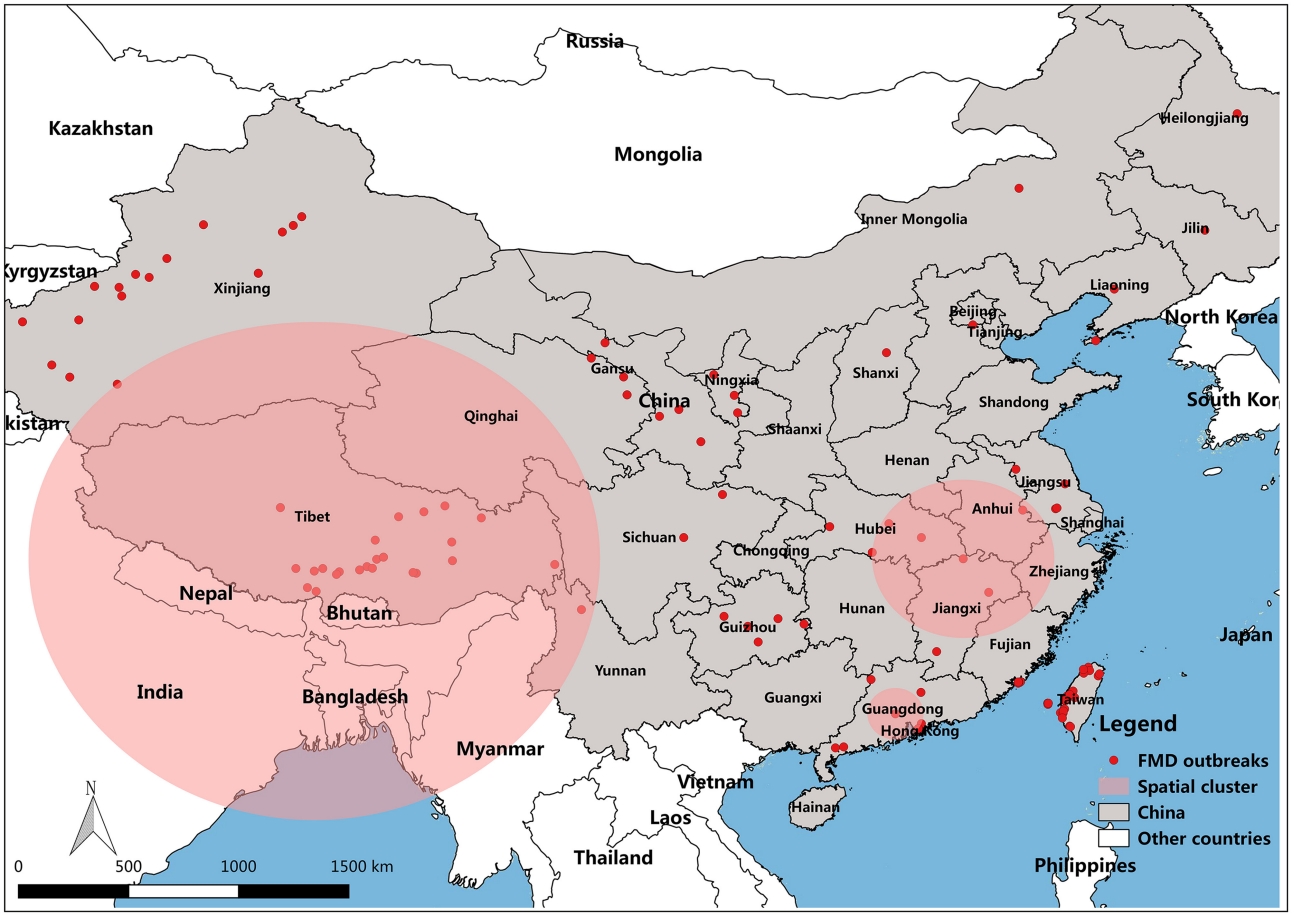
Geographical localization of FMD clusters in China.

At the same time, the distribution of FMD in China and its spatial and temporal effects were analyzed using the spatio-temporal scanning model. The space-time cluster data are shown in Figure 8 and Table 3. There were a total of 129 localities with 12,373 FMD cases contained in China identified as certain infection sources for the animals. The scan test identified three significant spatio-temporal clusters of FMD outbreaks (*P* < 0.001). The first was in Guangdong province with three localities. The center of the second cluster covered Wuhan, Jiangxi, and Anhui provinces with six FMD localities. The third and biggest cluster was located in Tibet, with 28 affected localities and a space-time cluster from 19 February 2013 to 15 December 2015, with 1,025 observed FMD cases.

**Table 3:** Characteristics of FMD clusters.

**Cluster ID**	**Radius (km)**	**Time frame**		**No. of cases included**	**No. of cases expected**	***P*-value**
3	121.49	2010/2/19	2010/2/22	2564	531.33	<0.001
6	372.39	2015/1/2	2016/11/15	843	60.68	<0.001
28	1171.63	2013/2/19	2014/12/15	1025	130.37	<0.001

### Directional Trend Analysis

**Figure 9:**
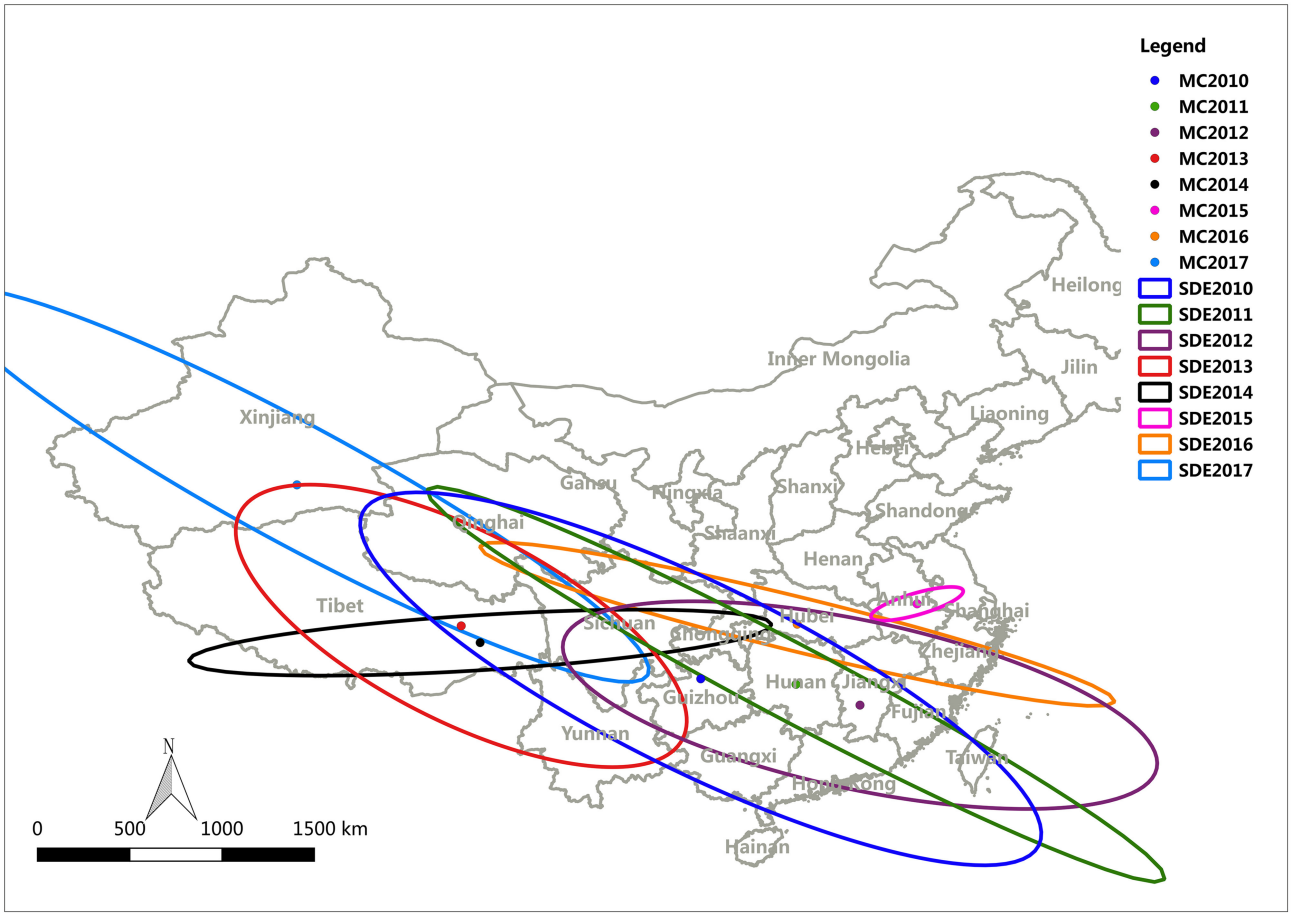
FMD mean center and standard deviational ellipse from 2003 to 2012.

To study the transmission and spread of FMD in China from 2010 to 2017, SDE was utilized to analyze the directional trend of FMD. The data for the SDE and distribution of epidemic regions caused by FMD are presented in Figure 9. The overall direction of FMD transmission was northwest. The rotation angle was maintained within range of 102.03–118.42, except for large-scale changes in 2014 and 2015, and the spatial pattern of FMD outbreaks displayed an overall northwest-southeast distribution (Table 4). The decreased rotation angle in 2012 and 2016 was manifest as a weakened northwest-southeast pattern. In 2013 and 2017, the rotation angle was increased to ~117°, which further strengthened the northwest-southeast spatial pattern. Considering 2010 as the starting point, the movement of mean center was southeast-southeast-northwest-northeast-southwest-southeast-northwest.

**Table 4:** The attributed values for the standard deviational ellipse.

**Year**	**Center X(^**°**^)**	**Center Y(^**°**^)**	**XstdDist**	**YStdDist**	**Rotation angle(^**°**^)**
2010	106.85	27.72	19.23	4.78	116.21
2011	111.71	27.44	21.28	1.91	117.85
2012	114.98	26.38	15.49	4.29	102.03
2013	94.63	30.42	12.69	4.85	117.05
2014	95.60	29.57	1.41	14.91	86.43
2015	117.90	31.55	0.55	2.45	79.93
2016	111.78	30.51	16.68	1.38	103.72
2017	86.24	37.62	20.37	3.04	118.43

### Phylogenetic Analysis of FMDV Genomes

**Figure 10:**
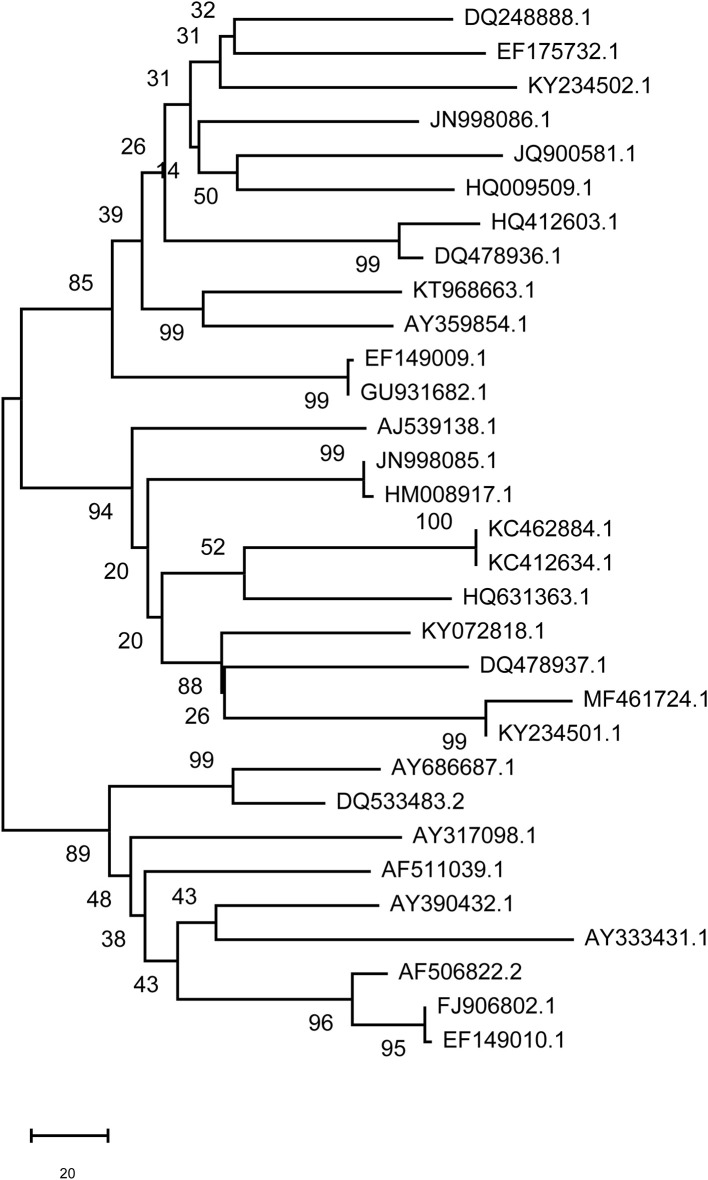
Phylogenetic analysis of FMDV genomes. N, not available.

The branches of a phylogenetic tree represent the genetic relationship, and the distance of the branches reveals, to a certain degree, the evolutionary relationships of the viral strains. The number between the branches of the phylogenetic tree indicates the reliability of the homology and the length of the branch indicates the degree of variation. The present phylogenetic tree divided FMDV in China into three pedigrees. The boot-strap of nearly all strain branches reached 100, indicating that the homology of these strains was very high (Figure 10). DQ533483.2 (Asia 1), AY390432.1 (Asia1), and AF511039.1 (O) were the earliest FMDV strain in China, appearing in 1958 according to NCBI data (Table S1). Homology analysis showed that the homology of these three FMDV strains with other strains ranged from 81 to 99%. There was high homology of the gene sequence between DQ533483.2 and AY390432.1, and the identity of these two strains was 98% by BLAST analysis of NCBI data. However, the identity between DQ533483.2 and AF511039.1 was 85%, which was the same as the result obtained by BLAST of AY390432.1 and AF511039.1. HQ412603.1 (2000, O) was identified as the virus variant for DQ533483.2 and AY390432.1, with 81.34 and 81.05% identity, respectively. For AF511039.1, AY686687.1 (2001, O) was the variant, and the identity between the two strains was 85.28%. The results show that 31 strains in China were in a high homology.

## Discussion

FMD is an acute and highly contagious disease. Despite intensive control and prevention efforts, FMD is still endemic and occurs extensively in many regions of the world (3, 32). In China, FMD was first reported in 1958 with three types found in Xinjiang Uygur Autonomous Region (serotypes O and A) and Yunnan province (serotype Asia 1) (33–35). Despite low mortality rates, 129 outbreaks of FMD have occurred in China since 2010 (36). With a goal of preventing the spread of FMD in the next few years using effective precautionary measures (37), it is important to describe and analyze spatial and temporal dynamics changes in FMD outbreaks.

Here, we explored the time distribution pattern of FMD outbreaks in China, and found obvious time patterns. The data highlight the importance of strengthening the surveillance of FMD outbreaks in the first half of the warm and rainy season. In China, FMD outbreaks have occurred in many provinces due to the weak centralized management of farms. The characteristics of spatial cluster analysis provide an effective way to identify the regions at risk (38). The spatial analysis indicated that the focus of prevention and control of FMD in China should be on Guangdong and Xinjiang provinces. The calculations of the spatial cluster and prevalence analyses were not comprehensive (39). As the methods of risk analysis have varied over space and time, retrospective permutation space-time scan statistics to provide complementary information are important and overcome the weaknesses of using single method (40, 41). Space-time analysis based on the number of cases and the geographical location of the epidemic regions revealed that the cluster patterns in Guangdong were significant between 19th and 22nd February, 2010.

FMD prevalence analysis revealed that FMD outbreaks were concentrated in the first half of the year. It seems likely that the warm and rainy weather in the spring and summer in most provinces of China is suitable for the spread of pathogenic microorganisms, including FMDV, while cold weather would inhibit FMDV replication and thus curb the spread of FMDV (42). However, Figure 2 and Table 1 show that the number of FMD cases in February and December reached 4,273 and 1,047, respectively. The affected localities in the FMD outbreak during February and December were mainly concentrated in Taiwan, Fujian, and Guangdong. Coastal cities in these regions experience a humid subtropical monsoon climate, characterized by a warm winter and hot summer. Even in the winter, FMDV spread in these areas would occur. However, other provinces, such as Xinjiang and Jiangsu, also experienced FMD outbreaks in winter. We surmise that the winter-related food shortage and lack of sunlight could reduce animal immunity (18, 43–45), contributing to FMDV transmission and increased risk of infection. In addition, the map of FMD cases (Figure 3) in the FMD prevalence analysis showed that Xinjiang and Tibet were the two provinces in China with a high frequency of FMD outbreaks. Frequent of outbreaks is considered that infectious animals in these two provinces are free-range, the relatively immune system is weak, and the prevention and control measures are insufficient.

The hotspots map constructed identified significant risk areas in each period. Further investigations in these areas would be worthwhile (46). In the affected localities, FMD cases and hotspots were similar, which indicates that the FMD hotspots reflect actual FMD outbreaks. In addition, the results of hotspots analysis are valuable for the surveillance and prevention of FMD, especially concerning immunization coverage (47, 48). FMD outbreaks reflect inadequate vaccine quality or implementation. Since the outbreak of FMD in 2010, the Ministry of Agriculture and Rural Affairs of China has implemented a system of immunization planning to provide compulsory immunization against FMD in the country. Based on different subtypes, FMDV animals were vaccinated with different vaccines (http://www.moa.gov.cn/gk/tzgg_1/tfw/201006/t20100606_1535849.htm). In Tibet, as an example, there were six hotspots in 2013. The outbreak and spreading of FMD in 2013 spurred measures to isolate infected areas. Disinfection, culling and non-harmful disposal of animals were jointly performed by the Ministry and the Tibet Autonomous Region government. In addition, Tibet initiated full coverage of compulsory immunization against major animal epidemics in 2014. Veterinary agencies in Tibet Autonomous Region have carried out supervision and guidance on the prevention and control of major animal epidemics in counties (cities and districts), and strengthened comprehensive measures for prevention and control of major animal epidemics (http://www.tibet.cn/cn/Instant/domestic/201811/t20181127_6423052.html).

After implementing quality standard improvement of FMD vaccine by the Ministry of Agriculture and Rural Affairs of China in 2013, no FMD hotspots were subsequently observed (Figure 4). In addition, among the common FMDV vaccines, including the O/Asia1/A serotypes trivalent inactivated vaccine, the O strain was selected from the most compatible strain, 0/MYA98/BY/2010. The Asia 1 strain used the classic Jiangsu strain, Asia1/JSL/ZK/06, and the type A strain used the Re-A/WH/09 strain.

Spatio-temporal analysis is an important tool to monitor changes of FMD epidemiology, identify high-risk regions and strengthen disease surveillance (49). In this study, Global Moran's I was established to describe global spatial autocorrelations. The spatial pattern of FMD outbreaks was significantly clustered. However, this approach is useless in identifying the exact location of clustered spatial patterns (50). Fortunately, the emergence of the LISA cluster map solved this problem. The high-high aggregation points of FMD outbreak from the LISA cluster map are consistent with the heat-dense points of FMD outbreaks from the heat map, which is located in Guangdong (2010) and Xinjiang (2017). The LISA cluster map revealed the absence of FMD clusters in 2015. However, in heat map, the Guangdong area remained a hot-dense point in which strengthened FMD prevention efforts were required. In space-time scan statistics, clusters center were found in Guangdong, Tibet and the junction of Wuhan, Jiangxi and Anhui. After comparing the spatial analysis and space-time analysis of FMD outbreaks, Guangdong was revealed to have the same cluster of the two. The spatial distribution pattern could determine if FMD outbreaks in Guangdong were a cluster distribution. In the spatio-temporal cluster analysis, the cluster in Guangdong was evident from 19th to 22nd February, 2010. The result of this spatial cluster may be due to problems in prevention and control measures, a temperature that was suitable for virus transmission during that time or other factors. The space-time cluster increases the accuracy of the regional division in the FMD surveillance zone. This allows other counties to establish an early warning system for the disease and reduce the loss of livestock husbandry.

The direction of the spread of FMD is another important factor as it provides a general direction for the integrated surveillance of FMD outbreaks and emphasizes defensive measures for the specific location of these clusters in the route of FMD outbreaks. SDE is a spatial statistical tool that is often utilized to depict the directional trend of the transmission and spread of disease. In this study, the change of the SDE rotation angle indicated that the spatial pattern of FMD outbreaks had a northwest-southeast distribution. In addition, almost all mean centers were located on or below the diagonal line between Tibet and Shanghai (northwest-southeast diagonal line), indicating that the cities where the average centers were located had a very important impact on the transmission of FMDV.

After analyzing distribution characteristics and transmission of FMD in China, a phylogenetic tree was constructed to analyze the homology and variation of FMDV genomes. We tried to discover the relationship between the homology and variation of FMDV genomes and an effective vaccine to control the spread of FMDV. The movement of the mean center of FMD revealed that most of the mean centers was located in the subtropical area south of the Huaihe River, except for Tibet, Xinjiang and Qinghai. Prevalence analysis showed that FMD outbreaks was focused on the first half year and most of the time in subtropical China was in a warm climate with sufficient rainfall and suitable temperature for the reproduction of pathogens. The mean centers moved back and forth along the northwest-southeast diagonal (Figure 9). Tibet, Xinjiang and Qinghai are in plateau areas with advanced grassland animal husbandry. The temperature and humidity in these regions are not conducive to FMDV reproduction, even though they are high outbreak areas of FMD. It might be that most of local residents in these provinces are nomads, cattle and sheep are in free-grazing, the immune system is weak, and the prevention and control measures are insufficient. There is always a risk of FMD outbreaks in these provinces of China, and measures to prevent and control FMD are needed.

There are many serotypes of FMDV, and the virus constantly mutates among different strains due to the high variation of serotype. In addition, FMDV vaccines do not provide excellent all-around protection against virus, since there are multiple routes of FMDV transmission along with low immunogenicity. A phylogenetic tree was constructed to study the prevalent status and the feature of gene subtype of FMDV strain and to prevent pandemic occurrence caused by varied FMDV strain variation in China. The homology and variation of the FMDV genome deduced from the phylogenetic tree will be helpful for the development of an effective vaccine. The phylogenetic analysis of the FMDV genome revealed HQ412603.1 (2000, O) and AY686687.1 (2001, O) as two virus variants for the three earliest FMDV strains that appeared in China in 1958. The study of FMDV, however, requires a long time and huge investment.

## Conclusion

This study analyzed FMD outbreaks and their spatio-temporal patterns in China from 2010 to 2017. As discovered by a directional trend analysis, the FMD outbreaks were oriented northwest-southeast. The trends of outbreaks and hotspots showed a decreasing trend from 2010 to 2017. To prevent the spread of FMD in this direction, more effective prevention and control measures should be taken before onset of the highly prevalent epidemic season. Analyses of spatial and space-time scan statistics revealed the 2010 FMD outbreaks from Guangdong as the most significant cluster. This type of analysis helps regulatory agencies to adopt timely defensive measures and establish a disease warning system to reduce the loss of livestock in response to specific locations of FMD outbreaks. FMDV in China can be divided into three pedigrees and the homology of these strains is very high. This provides clues for the development of FMD vaccines. In the future, a systematic post-vaccination sero-monitoring program can be developed based on FAO/EuFMD, to monitor sero-conversion and protective status.

## Data Availability Statement

Publicly available datasets were analyzed in this study. This data can be found here: http://empres-i.fao.org/eipws3g/#h=0.

## Author Contributions

BN and QC designed the study. YL, JW, and JC collected the data. BN, QC, MW, RL, QZ, and JC analyzed the data. All authors took part in interpreting the data and writing the manuscript.

### Conflict of Interest

The authors declare that the research was conducted in the absence of any commercial or financial relationships that could be construed as a potential conflict of interest.

## Supplementary Material

Table S1 Foot-and-mouth disease virus strain information.
